# A novel dual NLRP1 and NLRP3 inflammasome inhibitor for the treatment of inflammatory diseases

**DOI:** 10.1002/cti2.1455

**Published:** 2023-06-22

**Authors:** Callum AH Docherty, Anuruddika J Fernando, Sarah Rosli, Maggie Lam, Roland E Dolle, Manuel A Navia, Ronald Farquhar, Danny La France, Michelle D Tate, Christopher K Murphy, Adriano G Rossi, Ashley Mansell

**Affiliations:** ^1^ Centre for Innate Immunity and Infectious Diseases Hudson Institute of Medical Research Clayton VIC Australia; ^2^ Department of Molecular and Translational Sciences Monash University Clayton VIC Australia; ^3^ University of Edinburgh Centre for Inflammation Research Queen's Medical Research Institute, Edinburgh BioQuarter Edinburgh UK; ^4^ Department of Biochemistry and Molecular Biophysics Washington University School of Medicine St. Louis MO USA; ^5^ Hub‐Bio Strategic Advising Lexington MA USA; ^6^ Adiso Therapeutics Concord MA USA

**Keywords:** drug targets, inflammasome, inflammation, NLRP1, NLRP3, pulmonary inflammation

## Abstract

**Objectives:**

Inflammasomes induce maturation of the inflammatory cytokines IL‐1β and IL‐18, whose activity is associated with the pathophysiology of a wide range of infectious and inflammatory diseases. As validated therapeutic targets for the treatment of acute and chronic inflammatory diseases, there has been intense interest in developing small‐molecule inhibitors to target inflammasome activity and reduce disease‐associated inflammatory burden.

**Methods:**

We examined the therapeutic potential of a novel small‐molecule inhibitor, and associated derivatives, termed ADS032 to target and reduce inflammasome‐mediated inflammation *in vivo*. *In vitro*, we characterised ADS032 function, target engagement and specificity.

**Results:**

We describe ADS032 as the first dual NLRP1 and NLRP3 inhibitor. ADS032 is a rapid, reversible and stable inflammasome inhibitor that directly binds both NLRP1 and NLRP3, reducing secretion and maturation of IL‐1β in human‐derived macrophages and bronchial epithelial cells in response to the activation of NLPR1 and NLRP3. ADS032 also reduced NLRP3‐induced ASC speck formation, indicative of targeting inflammasome formation. *In vivo*, ADS032 reduced IL‐1β and TNF‐α levels in the serum of mice challenged i.p. with LPS and reduced pulmonary inflammation in an acute model of lung silicosis. Critically, ADS032 protected mice from lethal influenza A virus challenge, displayed increased survival and reduced pulmonary inflammation.

**Conclusion:**

ADS032 is the first described dual inflammasome inhibitor and a potential therapeutic to treat both NLRP1‐ and NLRP3‐associated inflammatory diseases and also constitutes a novel tool that allows examination of the role of NLRP1 in human disease.

## Graphical abstract

ADS032 represents the first described dual NLRP1 and NLRP3 inflammasome inhibitor, effective in reducing human inflammasome‐mediated inflammation *in vitro*. ADS032 also effectively reduces both pulmonary and systemic inflammasome‐induced inflammation. Taken together, ADS032 may provide an effective therapeutic strategy to reduce inflammasome‐associated inflammatory diseases.

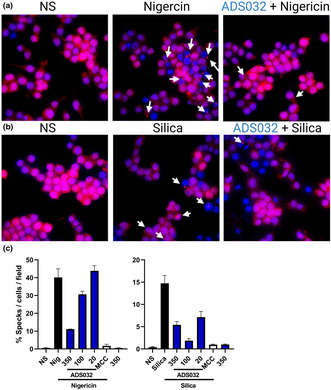

## Introduction

The NOD‐like receptor (NLR) family of innate cytosolic sensors recognises a diverse range of pathogen‐ and host‐derived factors. Upon activation, NLRP3 (NLR pyrin‐containing protein‐3) undergoes ATP‐induced catalytic ‘unfolding’ that allows recruitment of an adapter protein via homotypic pyrin domain interaction with apoptosis‐associated speck‐like protein containing a CARD (ASC).^1^, ^2^ ASC in turn recruits the cysteine protease caspase‐1 via CARD:CARD homotypic interaction, forming a multimeric oligomeric complex commonly referred to as a ‘speck’ in the cytosol of activated cells.^3^ Caspase‐1 undergoes autocatalytic cleavage, allowing proteolytic maturation of the prototypic inflammatory cytokines IL‐1β and IL‐18 into their bioactive forms, leading to subsequent secretion from the cell.^4^ Active inflammasomes also induce an inflammatory form of cell death termed pyroptosis.^5^ While NLRP3 is the most widely studied and characterised NLR sensor, there are other members of the NLR family capable of forming active inflammasome complexes. These include NLRP1 and NLRC4 (NLR CARD‐containing protein 4), in addition to other pattern recognition receptors such as absent in melanoma 2 (AIM2).^1^, ^2^ Non‐canonical inflammasome activation of caspase‐1 can also occur upon caspase‐11‐ and caspase‐8‐mediated recognition of intracellular lipopolysaccharide (LPS).^6^

Aberrant activation of NLRs is associated with the pathophysiology of a broad range of inflammatory diseases.^7^, ^8^, ^9^, ^10^, ^11^ NLRP3 gain‐of‐function mutations are associated with inheritable disorders such as cryopyrin‐associated periodic syndromes (CAPS: Muckle–Wells syndrome, familial cold autoinflammatory syndrome and neonatal‐onset multisystem inflammatory disease)^12^ and has also been associated with the pathophysiology of several complex multi‐organ diseases, notably neurodegenerative (Alzheimer's and Parkinson's disease), cardiovascular, metabolic (diabetes and obesity), pulmonary (chronic obstructive pulmonary disease (COPD) and asthma) diseases and cancer.^8^ We and others have also demonstrated a critical role for inflammasomes such as NLRP3 and NLRP1 in infectious diseases such as influenza A virus (IAV) and rhinovirus.^13^, ^14^, ^15^, ^16^, ^17^ Significantly, it is becoming increasingly evident that NLRP1 dysfunction is also associated with skin‐related genetic disorders such as NAIAD (NLRP1‐associated autoinflammation with arthritis and dyskeratosis), systemic sclerosis, multiple self‐healing palmoplantar carcinoma (MSPC) and familial keratosis lichenoides chronica (FKLC).^18^, ^19^, ^20^ Furthermore, similar to NLRP3, NLRP1 has increasingly been linked with neurodegenerative, pulmonary, metabolic, skin inflammatory and intestinal disease pathobiology.^21^

Current therapeutic treatments for cardiovascular disease and genetic NLRP3‐linked diseases such as CAPS are therapies targeting IL‐1β, including the neutralising monoclonal antibody canakinumab, the soluble IL‐1 receptor rilonacept or recombinant IL‐1 receptor antagonist anakinra.^22^ To date, pharmacological targeting of inflammasomes has focused upon NLRP3 activity, with the sulfonylurea NLRP3‐specific small‐molecule‐inhibitor MCC950 (also called CP‐456,773^23^) demonstrating considerable success in multiple *in vitro* and *in vivo* models of disease.^24^, ^25^, ^26^, ^27^ However, MCC950 has not progressed into late development, as patients treated in phase II clinical trials with CP‐456,773 for rheumatoid arthritis were found to have elevated serum liver enzyme levels.^25^ Several new small‐molecule NLRP3‐specific inhibitors, however, have undergone phase I/II clinical trials such as Inflazome's (purchased by Roche, 2020) brain‐penetrant Inzomelid (IZD174: NCT04086602), Somalix (IZD34: NCT04015076) for treatment of CAPS and IFM Tre's IFM2427 (obtained by Novartis and renamed DFV890; NCT04868968, NCT04382053) tested for CAPS efficacy and recently COVID‐19 pneumonia. Furthermore, Zydus Cadila (ZYIL1: NCT04972188) and NodThera (NT‐0796; ACTRN12621001082897) have initiated phase I safety trials with their MCC950‐derived molecules.^24^ Additionally, identification of a broad‐spectrum inflammasome inhibitor MM01 that disrupts ASC‐mediated inflammasome oligomerisation^28^ has raised the potential of targeting multiple inflammasome complexes. To date, there is no specific inhibitor described for NLRP1. Given the overlapping and complementary roles of both NLRP3 and NLRP1 in a wide variety of diseases, we sought to develop therapeutic targets to antagonise inflammasome activity.

We have previously described the sulfonylurea compound probenecid as inhibiting the NLRP3 inflammasome,^29^ reducing pulmonary inflammation following IAV challenge and providing protection against lethal IAV infection. We, therefore, developed small‐molecule compounds based on the structure of probenecid as potential inflammasome inhibitors. Structural modification and screening led to the identification of ADS032 as a dual NLRP1 and NLRP3 inhibitor that is effective in human and mouse primary cells and cell lines. In contrast to other sulfonylurea inhibitors, ADS032 directly binds to both NLRP1 and NLRP3, reducing IL‐1β secretion and maturation and blocking speck formation. ADS032 reduced inflammation in an *in vivo* model of LPS‐induced septic shock and reduced pulmonary inflammation in a mouse model of acute silicosis. Importantly, while we had previously found that ablating NLRP3 functionality with a potent inhibitor such as MCC950 had both positive and detrimental disease outcomes during IAV infection,^15^ we have found that mice treated with ADS032 at any time during IAV infection had increased survival and reduced pulmonary inflammation.

Thus, ADS032 represents the first reported NLRP1 inhibitor and is a potential therapeutic to treat NLRP1‐ and NLRP3‐associated inflammatory diseases. ADS032 is a novel tool to allow examination of the role of NLRP1 in human disease.

## Results

### ADS032 is a novel NLRP3 and NLRP1 inflammasome inhibitor

**Figure 1:**
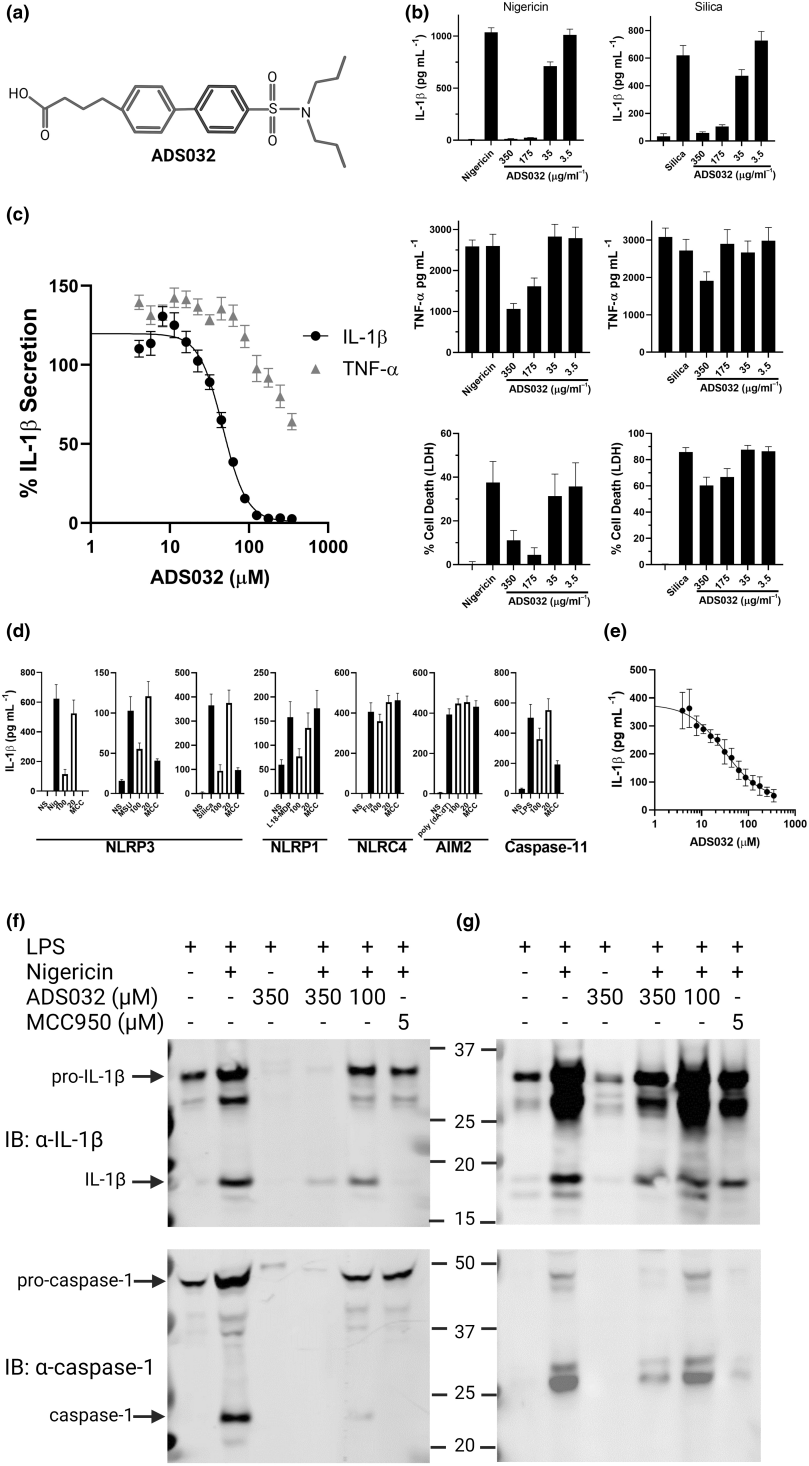
ADS032 is a novel NLRP3 inhibitor. **(a)** ADS032 structure. **(b)** Immortalised bone marrow‐derived macrophages (iBMDMs) were seeded at 4 × 10^5^ mL^−1^ prior to priming with LPS (100 ng mL^−1^) for 3 h. Media were removed 60 min prior to challenge and replaced with serum‐free media containing ADS032 (3.5–350 μm) as indicated, or DMSO control (0.8% v/v). Macrophages were stimulated with either nigericin (6 μm) or silica (250 μg mL^−1^) as indicated for 120 or 360 min respectively. Cellular supernatants were analysed for secreted IL‐1β, TNF‐α by ELISA or cell death by LDH assay as indicated. **(c)** iBMDMs were treated with a range of ADS032 concentrations for 60 min in serum‐free media prior to challenge with nigericin for 120 min. The results shown are representative of three independent experiments carried out in triplicate and presented as the mean ± SEM. Non‐linear regression analysis was performed, and the curve of the log [*M*] ADS032 versus the normalised response (variable slope) is presented. **(d)** LPS (3 h;100 ng mL^−1^)‐primed iBMDMs were pre‐treated with ADS032 (20 and 100 μg mL^−1^) or MCC950 (MCC: 5 μg mL^−1^) in serum‐free media for 60 min and treated with NLRP3 (nigericin, 6 μm, 120 min; monosodium urate (MSU), 250 μg mL^−1^, 6 h; and silica, 250 μg mL^−1^; 6 h), NLRP1 (L18‐MDP, 100 μg mL^−1^, 16 h), non‐canonical NLRP3 (transfected LPS *Escherichia coli* serotype 0111 B4, 2.0 μg mL^−1^, 16 h), AIM2 (poly dA:dT, 1 μg mL^−1^, 8 h) and NLRC4 (flagellin, 20 μg mL^−1^, 16 h) agonists as indicated. Secreted IL‐1β was assayed by ELISA and the results shown are representative of three independent experiments carried out in triplicate and presented as the mean ± SEM. **(e)** IC_50_ value of L18‐MDP (NLRP1) activity was determined as normalised IL‐1β secretion in ADS032‐treated iBMDMs. **(f)** BMDMs generated from wild‐type mice, and **(g)** PMA‐differentiated THP‐1 macrophages were primed with LPS for 3 h, treated with ADS032 (100 and 300 μm) or MCC950 (5 μm) where indicated for 60 min, then subsequently challenged with nigericin for 180 min. Cultured supernatants were assayed for secreted IL‐1β and caspase‐1 following 4–12% SDS‐PAGE, transfer to PVDF membrane and immunoblotting (IB) with indicated antibodies. The results shown are representative of three independent experiments.

In an endeavour to identify potential therapies for the treatment of inflammasome‐mediated diseases, we screened an in‐house bioactive compound library against immortalised mouse bone marrow‐derived macrophages (iBMDMs) pre‐treated with LPS and challenged with prototypic NLRP3 agonists nigericin and silica.^30^, ^31^ We identified a family of structurally related, putative NLRP3 antagonists that inhibited IL‐1β secretion, with ADS032 selected for further characterisation. ADS032 is a sulfonylurea compound (Figure 1a), which inhibited, in a concentration‐dependent manner, both ionophore‐ (nigericin) and crystalline‐ (silica) induced IL‐1β and LDH secretion from LPS‐primed iBMDMs, respectively, while LPS‐induced TNF‐α production was largely unaffected (Figure 1b). The half‐maximal inhibitory concentration (IC_50_) for nigericin‐induced NLRP3‐induced IL‐1β secretion was approximately 30 μm (Figure 1c).

We next examined the specificity of ADS032 against other inflammasome complexes. ADS032 and MCC950 inhibited IL‐1β secretion elicited by multiple NLRP3 agonists such as nigericin, silica and monosodium urate (MSU), but did not affect activation of non‐canonical inflammasome activity (LPS), AIM2 (poly(dA:dT)) or NLRC4 (flagellin) (Figure 1d). Critically, while ADS032 dose dependently inhibited IL‐1β secretion induced by the NLRP1 agonist L18‐MDP, MCC950 had no effect, highlighting that ADS032 is both an NLRP3 and NLRP1 antagonist. However, it has been reported that L18‐MDP is an NLRP3, rather than an NLRP1, agonist in a mouse model of challenge.^32^ Therefore, to determine whether L18‐MDP is a specific NLRP1 agonist, we found that L18‐MDP induces IL‐1β secretion from both WT and NLRP3‐deficient immortalised bone marrow‐derived macrophages, while nigericin‐induced secretion of IL‐1β is ablated in only the NLPR3‐deficient macrophages. This would suggest, therefore, that in murine macrophages at least, L18‐MDP‐induced IL‐1β secretion is not NLPR3 dependent. Importantly, ADS032 reduced L18‐MDP‐induced IL‐1β secretion in both WT and NLRP3‐deficient macrophages in a dose‐dependent manner (Supplementary figure 1). To further characterise ADS032 inhibitory function, we next demonstrated concentration‐dependent inhibition of L18‐MDP‐induced NLRP1 inflammasome activity with an IC_50_ of approximately 30 μm (Figure 1e), highlighting the additional pharmacologic effect of ADS032 on NLRP1 activation.

We next determined that ADS032 was targeting NLRP3 function by demonstrating that the amounts of caspase‐1 p10 and IL‐1β p17 (the matured and bioactive processed fragments of caspase‐1 and pro‐IL‐1β, respectively) were reduced in supernatants from ADS032‐treated iBMDMs, with the 100 μm dose reducing IL‐1β and caspase maturation without affecting NF‐κB responses in iBMDMs (Figure 1f) and human THP‐1 macrophages (Figure 1g). Similarly, treatment with MCC950 inhibited both caspase‐1 and pro‐IL‐1β processing as expected, suggesting that ADS032 affects the capacity of the inflammasome to become catalytically active.

Taken together, these findings clearly identify ADS032 as a novel, dual NLRP1 and NLRP3 inhibitor.

### ADS032 directly binds to NLRP1 and NLRP3

**Figure 2:**
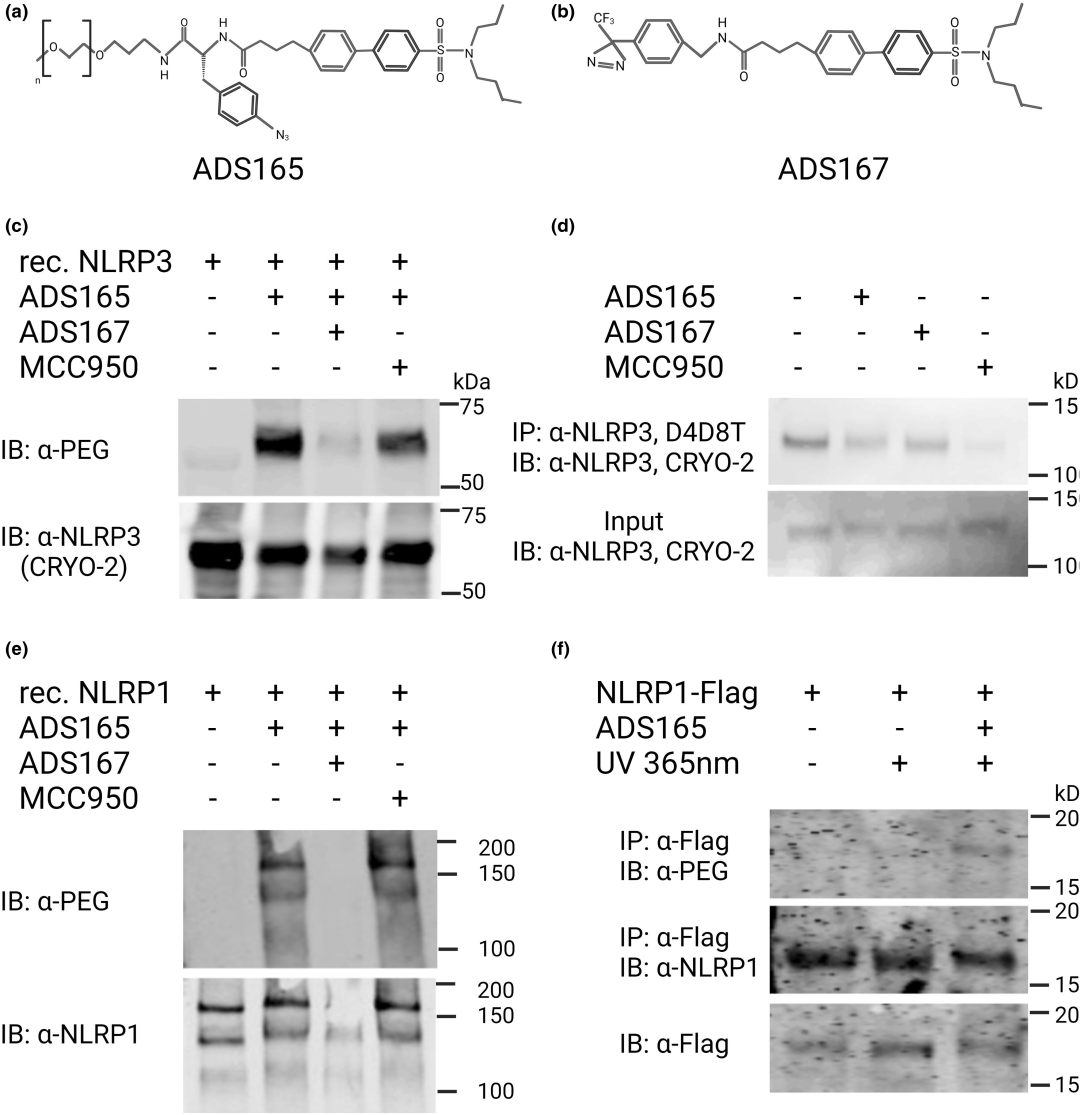
ADS032 directly binds to NLRP1 and NLRP3. **(a, b)** ADS165 and ADS167 structure. **(c)** Recombinant NLRP3 (2 μg) or **(e)** NLRP1 (2 μg) was co‐incubated where indicated with ADS167 or MCC950 for 20 min and then irradiated with UV 365 nm for a further 20 min. ADS165 was then co‐incubated for 20 min and UV treated for 20 min to cross‐link‐associated compound and protein. Protein was separated by SDS‐PAGE and immunoblotted (IB) with anti‐PEG to visualise ADS165‐linked protein. **(d)** iBMDM cells stably expressing NLRP3‐Flag were treated for 30 min with ADS165, ADS167 (1 mm) or MCC950 (50 μm) for 30 min, and irradiated with UV 365 nm for 30 min. Cells were lysed with buffer and immunoprecipitation (IP) of NLRP3 was performed with α‐NLRP3 (D4D8T) antibody. Levels of precipitated (IP) and total cellular lysate (Input) NLRP3 were determined by immunoblotting with α‐NLRP3 (Cryo‐2) antibody. **(f)** NLRP1‐Flag was expressed in HEK293T cells (1 × 10^6^) for 20 h and treated with ADS165 (1 mm) for 30 min where indicated, followed by UV 365 nm for a further 30 min. Cells were lysed with lysis buffer and NLRP1 immunoprecipitated with α‐Flag (M2) antibody. Proteins were separated by SDS‐PAGE and ADS165‐linked NLRP1 identified by immunoblot with α‐PEG, while total precipitated NLRP1 was visualised by immunoblot with α‐NLRP1 and total cell lysate by α‐Flag. The results shown are representative of three independent experiments.

To assess interaction with target proteins, a photo‐affinity labelling strategy^33^ was employed using two modified versions of ADS032: (1) containing an alkyne photoactivatable moiety linked with a 500‐Da *O*‐methyl polyethylene glycol (PEG) moiety to allow visualisation with anti‐PEG antibody (Figure 2a) termed ADS165 and (2) ADS032 linked with a diazirine photo‐affinity label on the hydroxy termini of ADS032 (Figure 2b) termed ADS167. Both photo‐affinity labels covalently cross‐link to molecular targets when exposed to ultraviolet light (365 nm). Despite these modifications, both ADS165 and ADS167 inhibited NLRP3‐induced IL‐1β secretion from iBMDMs (although with reduced efficacy as compared to ADS032), suggesting commensurate target engagement (Supplementary figure 2). Next, recombinant NLRP3 minus the leucine‐rich repeat region (molecular weight approximately 66 kDa), co‐incubated with ADS165 (ADS032 linked to PEG) and exposed to UV light was identified by immunoblot with α‐PEG antibody, suggesting that ADS165 was covalently linked to NLRP3 (Figure 2c). Pre‐incubation of NLRP3 with ADS167 and exposure to UV light inhibited the ability of ADS165 to label NLPR3, suggesting that both photo‐affinity labels are competing for the same binding site on NLRP3. Conversely, MCC950, which binds the Walker B domain of NLRP3,^34^, ^35^ did not inhibit ADS165 binding to NLPR3. This may be as a result of MCC950 not covalently binding NLRP3, and thus not being able to effectively block ADS032 from binding to NLRP3 (Figure 2c). Given this result, we sought to identify whether the ADS032 binding site may be within the NACHT domain of NLRP3. As described,^34^ the D4D8T NLRP3 antibody recognises residues around alanine 306, which is proximal to the Walker B motif within the NACHT domain of NLRP3. Immunoprecipitation of NLRP3 with this antibody from iBMDMs was reduced by pre‐treatment and photo‐labelling the cells with either ADS165 or ADS167 (Figure 2d). Consistent with previous reports, MCC950 also blocked NLPR3 immunoprecipitation in this assay.^34^ Importantly, cross‐linking of ADS165 to N‐termini Flag‐tagged NLRP3 did not affect the ability of anti‐Flag antibodies to recognise and immunoprecipitated NLRP3 from cell lysates, supporting the specificity of binding of ADS165 to NLRP3 (Supplementary figure 3a).

We next analysed the interaction of ADS032 with NLRP1. ADS165 was able to ‘tag’ recombinant full‐length Flag‐NLRP1 in whole cells (Figure 2e, second column) as determined by anti‐PEG immunoblot of anti‐Flag immunoprecipitated material. As with NLRP3, ADS167 blocked this interaction, while MCC950 had no effect. This suggests that the ADS032 moiety of ADS167 directly binds the NACHT domain of NLRP1, analogous to the observation for NLRP3. To further investigate this interaction, we immunoprecipitated ectopically expressed Flag‐tagged NLRP1 from cell lysates co‐incubated with ADS165 and exposed to UV light. As can be seen in Figure 2f, ADS165‐tagged NLRP1‐Flag was only isolated from cells exposed to both ADS165 and UV, consistent with the ADS032 moiety of the molecule directly interacting with NLRP1 *in vitro*. Finally, ADS165 specifically ‘tagged’ recombinant NLRP1 and NLRP3, but not a non‐specific protein in the extra‐cellular domain of recombinant interferon receptor 1 (IFNAR1; molecular weight approximately 67 kDa) (Supplementary figure 3b).

Overall, these results would suggest that ADS032 directly interacts proximal to the Walker B motif within the NACHT domain of both NLRP1 and NLRP3, thus inhibiting inflammasome activation and formation of each inflammasome complex.

### ADS032 inhibits ASC inflammasome oligomerisation

**Figure 3:**
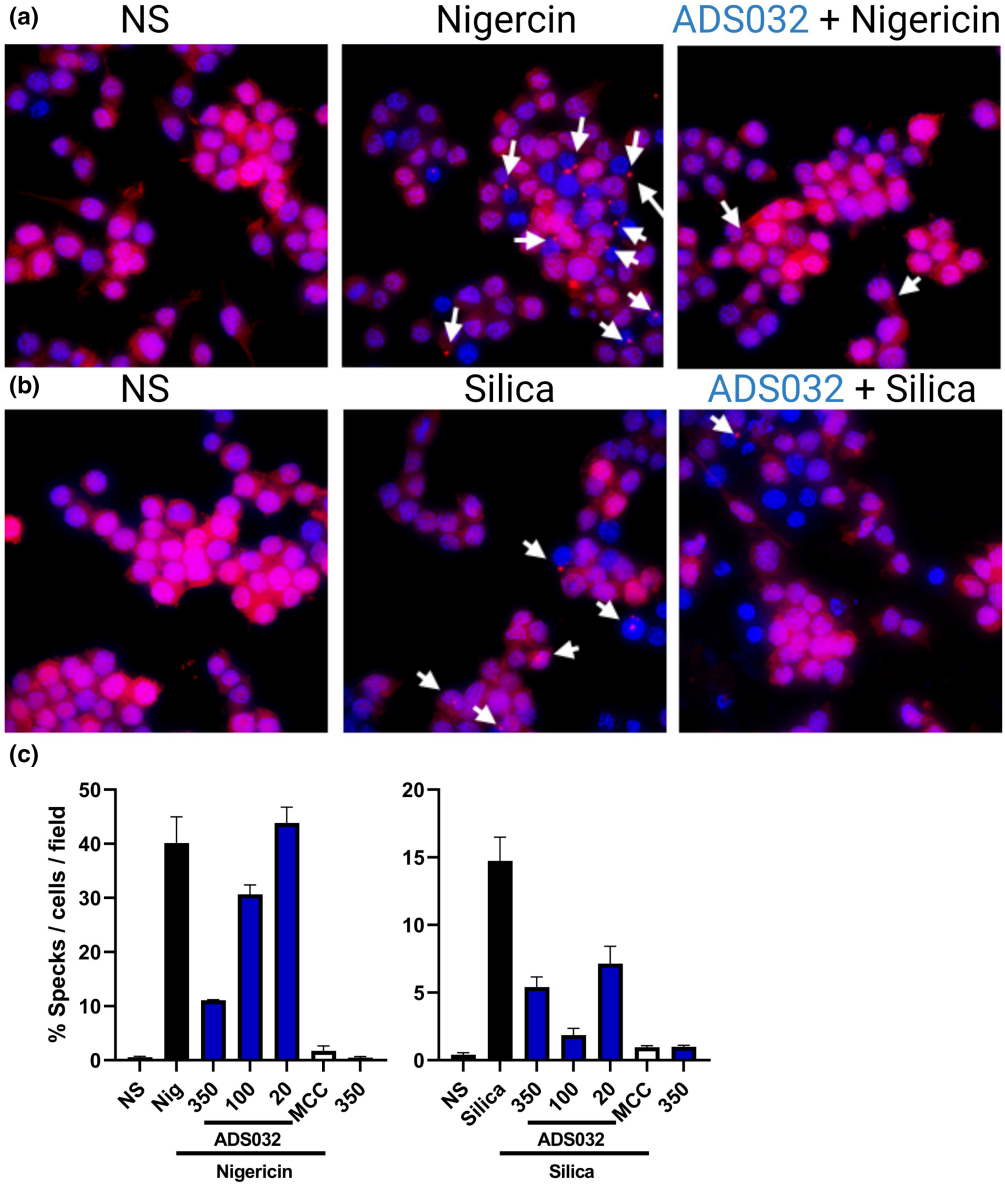
ADS032 dose dependently reduces NLPR3‐induced ASC oligomerisation. ASC‐tagged‐Cerulean (pseudo‐coloured Red) cells were treated or not with ADS032 (20–350 μm) or MCC950 (MCC: 5 μm) for 60 min and then challenged with either **(a)** nigericin (6 μm, 120 min) or **(b)** silica (250 μg mL^−1^, 5 h) before fixation. Cells were stained with Hoechst 33342 to identify cell nuclei (Blue). Images are flattened maximum‐intensity projections of three‐dimensional deconvolved *z* stacks. Specks were observed with confocal microscopy (magnification, ×630) and **(c)** the percentage of specks per field of view (nine fields per treatment group) were counted and compared to agonist‐treated cells. NS, not stimulated. Data presented are representative of three independent experiments.

NLRP3 activation results in the recruitment of ASC proteins and the formation of the high‐molecular‐weight oligomeric structure, the ASC speck, leading to the recruitment and subsequent catalytic activation of caspase‐1. Given our observations above, we determined whether ADS032 inhibited inflammasome complex formation. To visualise ASC speck formation, we used NLRP3‐deficient macrophages stably reconstituted with ASC‐cerulean and NLRP3‐Flag. Reconstituting these macrophages with the tagged ASC and NLRP3 obviates the need to induce NF‐κB priming prior to nigericin or silica activation. In the absence of inflammasome challenge, ASC appears diffuse throughout the cytosol (Figure 3a and b, left panel). However, upon nigericin (Figure 3a, middle panel) or silica (Figure 3b, middle panel) challenge, single intense fluorescent ASC foci, or specks, which are well‐characterised markers of inflammasome activation, were observed in the cytosol. The number of observed ASC specks was significantly reduced in cells that were pre‐treated with ADS032 (right panel) prior to NLRP3 agonist stimulation. Quantification of the number of specks detected per field of view highlights that ADS032 concentration dependently reduced NLRP3‐induced speck formation (Figure 3c) in response to nigericin and silica challenge, respectively, as was observed with MCC950. Taken together with the direct NLRP3‐binding activity, these results support the hypothesis that ADS032 directly inhibits formation of NLRP3‐mediated ASC speck formation.

### ADS032 is an effective NLRP1 and NLRP3 antagonist in human macrophages and epithelial cells

**Figure 4:**
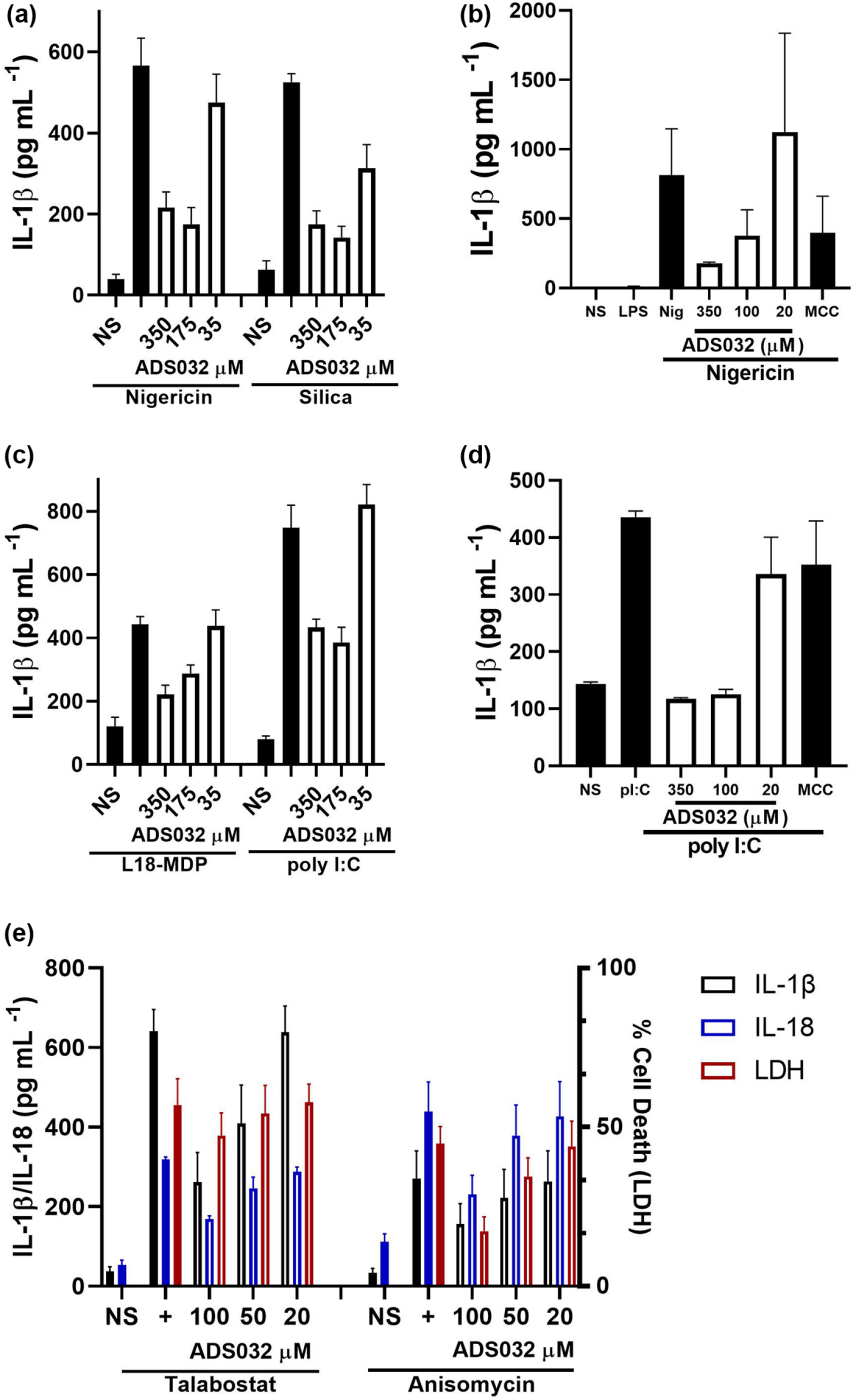
ADS032 effectively inhibits NLRP1 and NLRP3 inflammasome activity in human macrophages and bronchial epithelial cells. PMA‐differentiated THP‐1 cells primed with LPS (100 ng mL^−1^) for 3 h, pre‐treated or not with ADS032 (35–350 μm) for 60 min and then challenged with **(a)** NLRP3 agonists nigericin (6 μm) or silica (250 μg mL^−1^) for 120 or 360 min, respectively, or **(c)** NLPR1 agonists L18‐MDP (100 μg mL^−1^) or transfected poly I:C (200 ng mL^−1^) for 16 or 8 h respectively. **(b)** Human monocyte‐derived macrophages were treated for 3 h with 50 pg mL^−1^ LPS, treated with ADS032 (20–350 μm) or MCC950 (MCC; 5 μm) in serum‐free media for 60 min, then challenged with nigericin (6 μm) for a further 120 min. Bronchial epithelial cells obtained from normal patients were treated with ADS032 as indicated for 60 min and **(d)** treated with poly I:C (200 ng mL^−1^) for a further 8 h or **(e)** talabostat (2.5 μm; 24 h) and anisomycin (2.0 μm; 8 h). Cultured supernatants were assayed for secreted IL‐1β by ELISA. The results shown are the pooled data of three independent experiments carried out in triplicate and presented as the mean ± SEM.

Given the interest in therapeutically targeting NLRP3 in human disease, we examined the inflammasome inhibitory activity of ADS032 in human cells. Differentiated human THP‐1 macrophages were treated with ADS032 and assayed for IL‐1β secretion following NLRP3 activation with nigericin and silica. ADS032 concentration dependently reduced NLRP3‐mediated IL‐1β secretion in response to these activators (Figure 4a). ADS032 also concentration dependently inhibited nigericin‐induced NLRP3 activity in primary human monocyte‐derived macrophages (Figure 4b).

While NLRP3 agonists are generally convergent between mouse and human cells, NLRP1 is unique in the divergence of its agonists and isoforms between human and mouse cells.^36^ Human THP‐1 macrophages challenged with L18‐MDP (which has been demonstrated to activate NLRP1 in both human and mouse macrophages^37^) display reduced IL‐1β secretion when pre‐treated with ADS032 (Figure 4c). However, as L18‐MDP may not be a specific NLRP1 agonist,^38^ poly I:C was tested as a specific agonist. ADS032 concentration dependently inhibited poly‐I:C‐induced IL‐1β secretion in both human THP‐1 and primary human bronchial epithelial cells (pBECs) (Figure 4c and d). Interestingly, we found that pBECs endogenously express pro‐IL‐1β and do not require priming prior to inflammasome challenge (Supplementary figure 4). Consistent with our previous data, MCC950 had no effect on pBECs activated with poly I:C (Figure 4d). We further confirmed that ADS032 dose dependently inhibited IL‐1β, IL‐18 and cell death (LDH) following challenge with the well‐characterised NLRP1 agonist talabostat,^39^ and the recently described ribotoxic stress NLPR1 agonist, anisomycin^40^, ^41^ (Figure 4e). These results confirm that ADS032 inhibits NLRP1 inflammasome activation in both human macrophage and epithelial cells.

Taken together, these findings identify ADS032 as the first dual NLRP1 and NLRP3 antagonist, and, to our knowledge, the first described specific NLRP1 inhibitor active in both mouse and human cells.

### ADS032 is a rapid and stable inhibitor of NLRP3 activity

**Figure 5:**
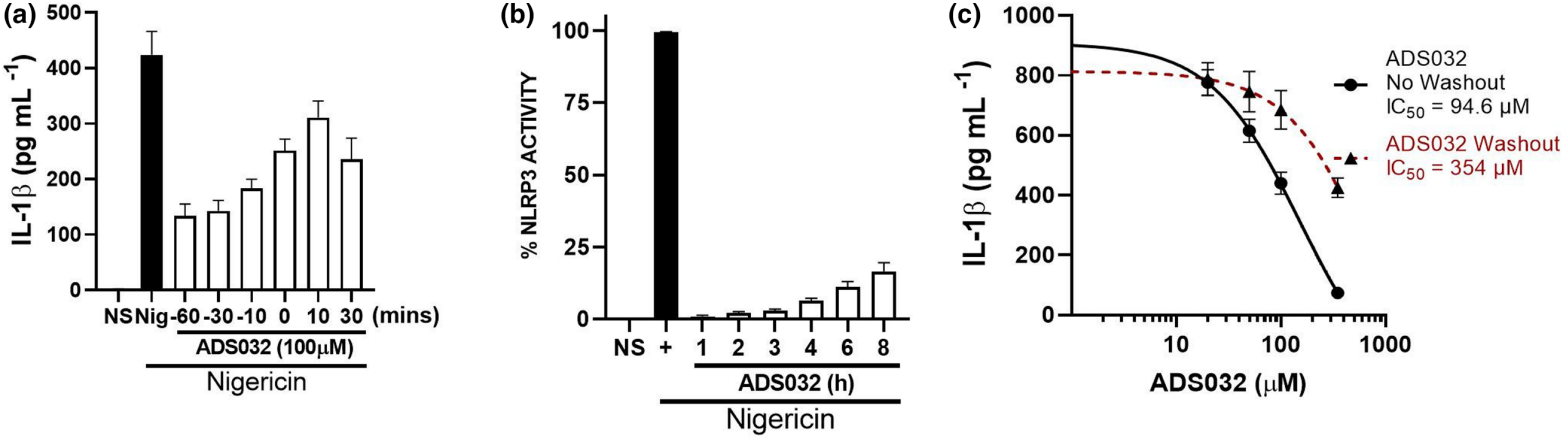
ADS032 is a rapidly acting, long‐lasting, reversible NLRP3 inhibitor. LPS (100 ng mL^−1^)‐primed iBMDM cells (2 × 10^5^ mL^−1^) were **(a)** treated with ADS032 (100 μm) prior (i.e. −10 to −60 min), simultaneously (i.e. 0 min) or post (i.e. 10 or 30 min) nigericin challenge, as indicated. **(b)** ADS032 (350 μm) was administered to primed iBMDMs for between 1 and 8 h prior to nigericin challenge for a further 120 min. **(c)** Primed iBMDMs were treated with ADS032 (20–350 μm) in the final 60 min of LPS priming and subsequently stimulated with nigericin for a further 2 h. ADS032 was either left on cells (No Washout) or removed (Washout) for 1 min prior to nigericin challenge. Secreted IL‐1β was assessed by ELISA and the data are representative of three independent experiments conducted in triplicate and presented as the mean ± SEM. Non‐linear regression analysis was performed **(c)**, and the curve of the log [*M*] ADS032 versus the normalised response (variable slope) is presented. The results shown are representative of three independent experiments carried out in triplicate and shown as the mean ± SEM.

To better understand the kinetics of ADS032 inhibition, iBMDMs were primed with LPS and then treated with ADS032 prophylactically (i.e. between 10 and 60 min prior to nigericin challenge), simultaneously with nigericin or therapeutically (i.e. up to 30 min after nigericin challenge). As can be seen in Figure 5a, ADS032 was effective in reducing nigericin‐induced IL‐1β secretion, even up to 30 min after nigericin stimulation, suggesting that ADS032 rapidly inhibits NLRP3 inflammasome activity, potentially even after nigericin‐induced activation. To determine the post‐treatment inhibition of NLRP3 by ADS032, LPS‐primed iBMDMs were treated with ADS032 and then exposed to nigericin and assayed for IL‐1β between 1 and 8 h post‐ADS032 treatment. ADS032 was effective at inhibiting nigericin‐induced IL‐1β secretion, even up to 8 h post‐treatment, suggesting that the pharmacologic effect of ADS032 is stable for prolonged periods (Figure 5b). Given the rapidity and stability of ADS032 inhibition, we next determined whether ADS032 covalently bound its target. In LPS‐primed iBMDMs, ADS032 robustly inhibited nigericin‐induced IL‐1β secretion (Figure 5c), as previously described. However, when ADS032 was washed out prior to nigericin challenge, its inhibition was decreased by approximately fourfold, with a shift of its apparent IC_50_ value from 44 to 173 μm, highlighting that ADS032 is a reversible inflammasome inhibitor.

These results demonstrate that ADS032 is a rapid, stable and reversible inflammasome inhibitor.

### ADS032 inhibits NLRP3 *in vivo* and reduces acute silicosis‐associated pulmonary inflammation

**Figure 6:**
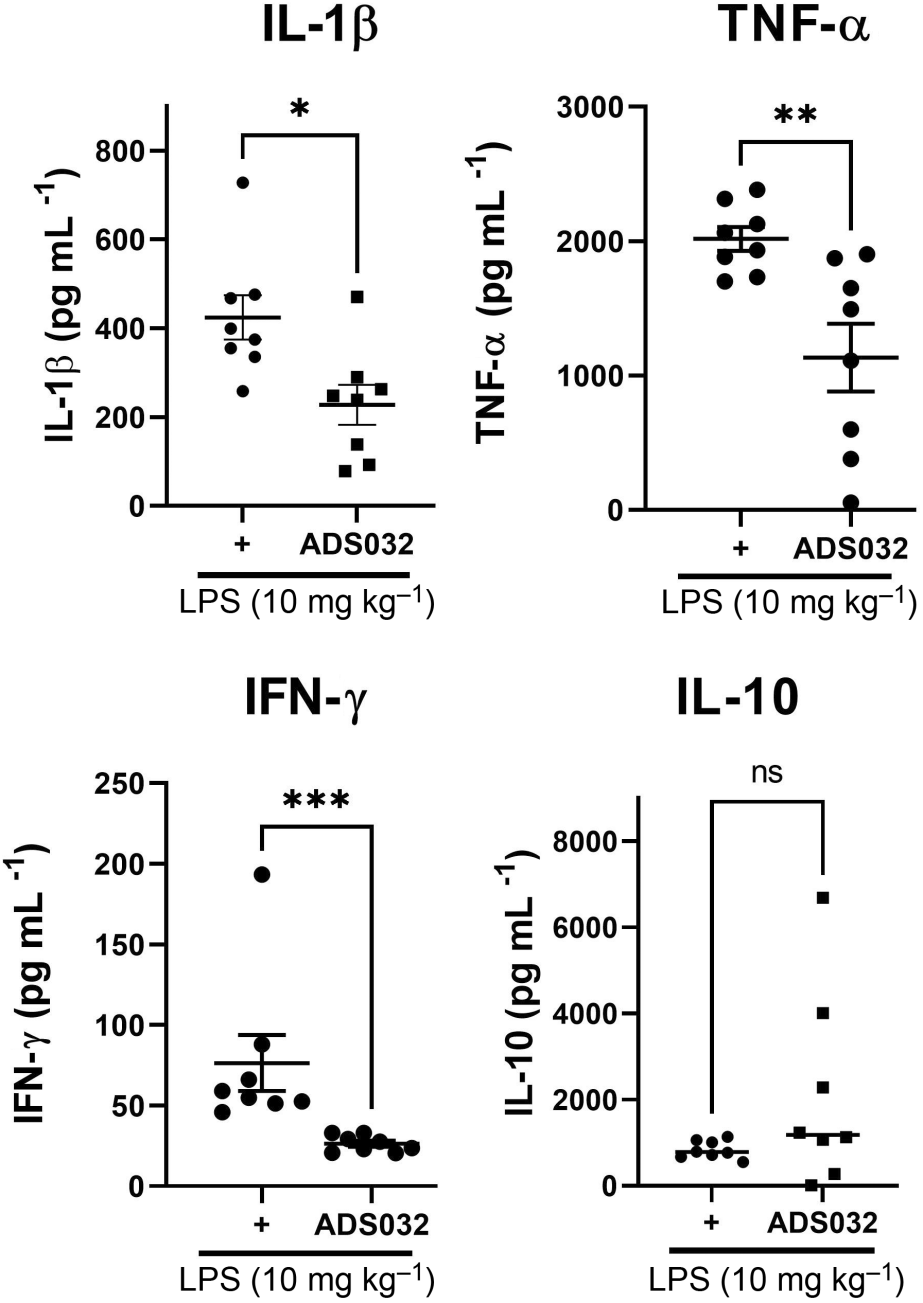
ADS032 reduced systemic inflammation *in vivo*. Serum levels of C57Bl/6 wild‐type mice (*n* = 8; 4 male/4 female per group) pre‐treated with ADS032 (200 mg kg^−1^) or vehicle control (methylcellulose) for 60 min prior to LPS challenge (10 mg kg^−1^, i.p.) for 120 min demonstrated reduced levels of IL‐1β, TNF‐α and IFN‐γ, but not IL‐10, as determined by ELISA or cytokine bead array. Data are presented as representative of two independent experiments. *P < 0.05, **P < 0.01 and ***P < 0.001, ns = not significant by the Mann–Whitney test.

Animal models of NLRP3‐mediated inflammation were next employed to assess the ability of ADS032 to block inflammation *in vivo*. Inflammation induced by i.p. administration of LPS has previously been shown to be NLRP3 dependent.^42^, ^43^ Mice pre‐treated i.p. with ADS032 for 60 min prior to i.p. LPS challenge (10 mg kg^−1^) display significantly reduced levels of circulating inflammatory cytokines IL‐1β, TNF‐α and IFN‐γ, while IL‐10 was not affected (Figure 6).

**Figure 7:**
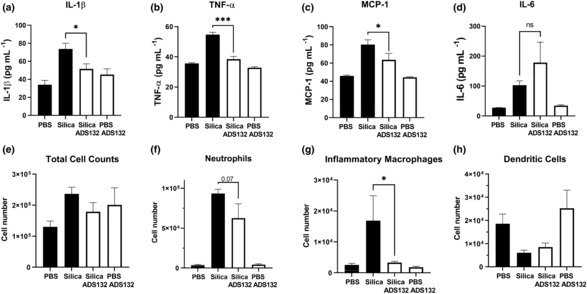
ADS032 reduces lung inflammation in an acute model of silicosis. Wild‐type C57Bl/6 mice (*n* = 6 per treatment group) received PBS alone, silica (50 mg kg^−1^), silica (50 mg kg^−1^) mixed with ADS132 (40 mg kg^−1^) in PBS or ADS0132 alone (40 mg kg^−1^) via intranasal inoculation. Twenty‐four hours later, mice were killed and bronchial alveolar lavage fluid was harvested. Proinflammatory cytokines **(a)** IL‐1β, **(b)** TNF‐α, **(c)** MCP‐1 and **(d)** IL‐6 were examined by ELISA or cytokine bead array. Total numbers of **(e)** leukocytes in BALF were determined by viable cell counts, **(f)** Ly6G^+^ neutrophils, **(g)** Ly6C^+^ inflammatory macrophages and **(h)** CD11c^+^ I‐Ab^high^ dendritic cells were determined by flow cytometry. The data presented are representative of two independent experiments and are the means ± SEM. *P < 0.05, ***P < 0.001, ns = not significant. One‐way ANOVA (multiple comparisons).

The efficacy of ADS032 in silica‐induced pulmonary inflammation, an acute model of human silicosis, was next tested. Clinical syndrome acute silicosis is characterised by rapid immune cell influx into the exposed lung and production of proinflammatory cytokines, including IL‐1β and chemokines.^44^ An *O*‐methyl PEG (550 Da)‐modified analogue of ADS032 that has enhanced solubility and a similar NLRP3 IC_50_, termed ADS132 (IC_50_ approximately 40 μm; Supplementary figure 5), was used in this experiment. Mice were intranasally instilled with either PBS, silica, silica simultaneously with ADS132 or ADS132 alone, and sacrificed at 24 h post‐instillation. Pulmonary inflammation in the mice was then examined by assaying inflammatory cytokines in bronchial alveolar lavage fluid (BALF). IL‐1β, TNF‐α and MCP‐1 inflammatory cytokine levels were significantly reduced in mice treated with ADS132 (Figure 7a–c), whereas IL‐6 levels were not affected, indicative overall of a reduction in lung inflammation (Figure 7d). Consistent with this, flow cytometric analysis of BALF cells illustrated that ADS132 treatment elicited a non‐significant reduction in neutrophils and a significant reduction in macrophages in the BALF, while dendritic cells remained slightly reduced in the silica‐treated mice (Figure 7e–h). These results highlight that ADS032 effectively reduced pulmonary inflammation *in vivo* in a model that is NLRP3 dependent.

### ADS032 is an effective treatment against IAV‐induced pulmonary inflammation and disease severity

We have previously demonstrated that MCC950 protects against the severity of influenza A virus infection and significantly reduces pulmonary inflammation when dosed 3 days post‐infection.^15^ Conversely, MCC950 treatment at 1 day after IAV infection renders mice hypersusceptible to even low‐dose IAV infection, consistent with disease outcomes in NLRP3‐deficient mice.^13^, ^16^ These studies highlighted that NLRP3 activity has a dual role during IAV infection, protective early in infection, but detrimental later, contributing to the ‘cytokine storm’ characteristic of lethal IAV infections.^45^, ^46^ NLRP3‐targeted therapies may thus require dosing that will allow an effective immune response during infection to reduce viral burden while lessening chronic, detrimental inflammation later in infection.

**Figure 8:**
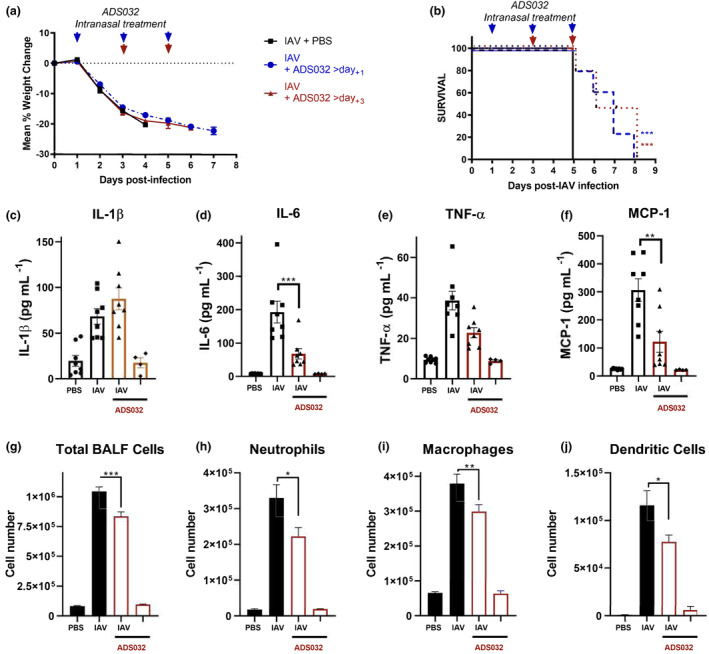
ADS032 therapeutically protects mice from severe IAV disease and reduces pulmonary inflammation. Groups of male and female C57Bl/6 mice (*n* = 8 per group) were intranasally challenged with HKx31 (10^5^ PFU) IAV. Mice were treated intranasally with PBS (IAV) or ADS032 (20 mg kg^−1^) from either day 1 or day 3 post‐infection and every 48 h thereafter. Uninfected mice (*n* = 4 per group) treated with PBS or ADS032 were included for comparison. **(a)** Mice were weighed daily and results were expressed as the mean ± SEM. **(b)** Survival curves are shown. ***P < 0.001. Mantel–Cox log‐rank test. Groups of mice, as described above, were also challenged with HKx31 (10^5^ PFU) and treated with ADS032 (20 mg kg^−1^) every 48 h from day 1. On day 4 post‐infection, mice were killed. Proinflammatory cytokines **(c)** IL‐1β, **(d)** IL‐6, **(e)** TNF‐α and **(f)** MCP‐1 were determined by ELISA or cytokine bead array in BALF. Total numbers of **(g)** leukocytes in BALF were determined by viable cell counts, and **(h)** Ly6G^+^ neutrophils, **(i)** total CD11c^+^ I‐Ab^low^ macrophages and **(j)** CD11c^+^ I‐Ab^high^ dendritic cells were determined by flow cytometry. The results shown are representative of two independent experiments and presented are the means ± SEM from eight mice in IAV/IAV‐ADS032 groups, and four mice in PBS/ADS032 groups. *P < 0.05, **P < 0.01 and ***P < 0.001. One‐way ANOVA.

We determined the efficacy of ADS032 in the mouse‐lethal IAV challenge model. Mice were intranasally challenged with a lethal dose (10^5^ PFU) of HKx31 (H3N2) in PBS on day 0. HKx31 is an IAV relative to PR8 derived from a human isolate from the deadly Hong Kong pandemic of 1968. Unlike the mouse‐adapted PR8 strain, HKx31 is similar to many pathogenic IAV strains such as H5N1 and H7N9 in its ability to infect macrophages.^47^, ^48^, ^49^ Mice were subsequently intranasally dosed with ADS032 or PBS alone on either days 1, 3 and 5 or days 3 and 5 post‐IAV infection as indicated. IAV‐infected mice treated with PBS display rapid weight loss (Figure 8a, black squares) and lethality (Figure 8b, solid line) within 5 days post‐IAV challenge. It should be noted that treatment with ADS032 was withdrawn after day 5 because of ethical considerations. Consistent with our previous observations with MCC950, mice treated with ADS032 from day 3 post‐challenge display delayed or reduced weight loss (Figure 8a, red triangles) and significantly reduced mortality (Figure 8b, red dotted line), highlighting that targeting NLRP3 protects against detrimental inflammation late in infection. Critically, however, when mice were treated with ADS032 from day 1 post‐IAV challenge, mice display reduced weight loss (Figure 8a, blue circles) and protection against IAV mortality (Figure 8b, blue dotted line), highlighting that ADS032 is protective when given either early or late during infection, in stark contrast to our previous observations with MCC950.

Having established that ADS032 protects against lethal IAV challenge regardless of when administered, we next examined the effect of ADS032 treatment upon pulmonary inflammation. Mice treated from day 3 post‐IAV challenge with ADS032 did not display reduced IL‐1β concentrations (Figure 8c) in BALF, there was a reduction in IL‐6, TNF‐α and MCP‐1 BALF concentrations (Figure 8d–f), and reduced total cellular infiltrates in the lungs (Figure 8g), characterised by significantly reduced neutrophils (Figure 8h), macrophages (Figure 8i) and dendritic cells (Figure 8j). Importantly, treatment with ADS032 did not induce any noticeable pulmonary inflammation itself when compared to PBS treatment alone.

These results demonstrate that ADS032 can suppress pulmonary cytokines and cellular infiltrates following IAV challenge, providing protection from mortality associated with severe IAV infection. This suggests that ADS032 could be used to reduce the inflammatory burden associated with pulmonary inflammation and infections.

## Discussion

In this study, we describe ADS032 as the first potent, dual and direct inhibitor of both NLRP1 and NLRP3, effective in both human and mouse cells *in vitro* and *ex vivo*, and also effective in reducing inflammation *in vivo*. ADS032, therefore, represents a novel tool to further pharmacologically investigate the role of NLRP1 and NLRP3 in disease biology, with the potential for translation into therapeutic applications to reduce the inflammatory burden in NLRP1‐ and NLRP3‐associated diseases.

While a number of NLRP3‐specific inhibitors have been described, a common characteristic feature among several, such as glyburide,^50^ CP‐456,773,^23^ MCC950,^42^ OLT1177^51^ and JC‐171,^52^ is a central sulfonylurea group. Indeed, recent structural studies of NLRP3 in complex with MCC950^53^, ^54^ identified that MCC950 interacted with five subdomains within the NACHT domain, with the central sulfonylurea group located on the backside of the Walker A motif in NLRP3, close to the side chains of alanines within the G_226_AAGIGKT motif, providing a potential mechanism of action for other sulfonylurea‐containing inhibitors. Interestingly, the Walker A domain of NLRP1 is the most similar among NLRs to that of NLRP3, comprising G_334_AAGIGKS. Our findings suggest that analogous to MCC950, ADS032 directly binds to the NACHT domain of NLRP3 and likely the same region in NLRP1, to inhibit activation of the complex and subsequent oligomerisation of the catalytically active NLRP/ASC/caspase‐1 complex. Future structural studies will be required to ascertain how ADS032 is able to bind and inhibit both NLRP1 and NLRP3, while these other sulfonylurea‐based compounds do not.

Small‐molecule targeting of inflammasome‐driven disease, while not currently clinically available, presents some advantages over the current use of targeted IL‐1β biologics such as anakinra, riloncept and canakinumab, which have been associated with the development of autoantibodies and increased risk of infections.^55^, ^56^ NLRP‐targeted molecules afford the opportunity to not only target IL‐1β maturation but also inhibit IL‐18 and pyroptosis, which also play pathophysiological roles in disease. While reducing inflammation is critical to improved disease outcomes, inflammation plays a key role in maturing the adaptive immune response. Other aspects of inflammation contribute to initiating tissue repair and a return to homeostasis, as evidenced by biologics‐treated patients suffering recurrent infections. In evidence of this, we and others found in models of inflammation that NLRP3‐deficient mice^13^, ^15^, ^16^ or potent inhibition of NLPR3 with MCC950 came at the cost of constraining the inflammatory response and inducing an ‘immunocompromised’ state. Indeed, an inflammatory ‘balance’ is required in the immune response to infection, as we have demonstrated in the case of severe IAV infection of mice. Thus, NLRP3‐mediated inflammation is both beneficial early, but detrimental later, contributing to pathophysiology and poor disease outcome. Indeed, the recent description of NLRP3 activity contributing to SARS‐CoV‐2 disease and potential markers of poor disease outcome,^57^, ^58^ further highlights the delicate balance of inflammasome activation required. As such, it would appear that potently targeting the inflammasome would require knowing ‘when’ to treat so as to only remove the detrimental NLRP3 inflammation and not the protective aspects. The studies herein suggest that ADS032 treatment elicits a protective environment by ‘balancing’ the inflammatory response, reducing the magnitude of inflammation to moderate the collateral damage that would lead to severe disease, but maintaining enough inflammation to regulate protective inflammatory processes. As such, we believe ADS032 represents a therapeutic intervention strategy without compromising the protective inflammatory environment required for homeostatic regulation.

To the best of our knowledge, ADS032 is the first characterised dual NLRP1 and NLRP3 inhibitor and, critically, the first identified NLRP1 inhibitor. Given the growing recognition and awareness of the role of NLRP1 in human disease, the lack of pharmacological targeting of NLRP1 has impeded the capacity to investigate and characterise the role of NLRP1 in pre‐clinical models and human disease. Furthermore, the divergent role of NLRP1 in mouse and human biology, owing to NLRP1 isoforms and vagaries between mouse strains and specificity of NLRP1 ligand recognition between mammalian species,^36^ has significantly hampered efforts to explore and characterise the NLRP1 biology. Indeed, in the context of this study, it should be noted that our animal model of IAV challenge should be viewed as an NLRP3‐mediated model of disease as no NLRP1 agonists to date has been recognised or described to induce NLRP1 activity in rodents. Importantly, however, our studies have demonstrated that ADS032 consistently inhibited the proinflammatory effects of multiple NLRP1 agonists (i.e. L18‐MDP and poly I:C) in both mouse and human cells *in vitro*, suggesting that ADS032 may be an excellent pharmacological agent to use as a pan‐species tool and agonist inhibitor. Indeed, the NLRP1 inflammasome has been suggested to play a dichotomous role in human diseases, functioning to either attenuate or augment miscellaneous biological processes in a tissue‐specific manner. The capacity of ADS032 to inhibit human NLRP1 function may allow the dissection of the role of NLRP1 in specific tissues and cell types in models of human disease.

While inflammasome inhibitors such as MCC950, NT‐0167, Cy‐09, OLT1177 and JC‐171 have highly effective and specific effects on NLRP3 activity, given the growing awareness of the association of both NLRP1 and NLRP3 inflammasome‐associated inflammation in many of the diseases that inflict a high global health burden, the capacity of ADS032 as a dual inflammasome inhibitor that acts to ‘rebalance’ inflammation may have widespread therapeutic applications.

## Methods

### Cell lines

Immortalised wild‐type C57BL/6 bone marrow‐derived macrophages (iBMDMs) and NLRP3‐deficient iBMDMs reconstituted with NLRP3‐Flag and ASC‐Cerulean (termed ASC‐cerulean iBMDMs) were grown in DMEM supplemented with 10% heat‐inactivated FBS and 2 mm glutamine supplied by Thermo Fisher (Melbourne, Australia). Bone marrow cells from C57BL/6 wild‐type mice were differentiated for 7 days in DMEM supplemented with 10% (v/v) FCS and 1% (v/v) penicillin/streptomycin solution (Thermo Fisher) and macrophage colony‐stimulating factor (20% v/v L929 mouse fibroblast supernatant).

Primary human airway cells^59^ were obtained from normal subjects who were non‐smokers or had not smoked for > 15 years; none had a diagnosis of asthma or COPD (normal FEV_1_ measurements). Studies were approved by the Monash Health and Monash Medical Centre Human Research Ethics Committee, consent was obtained from all subjects and studies were conducted in accordance with the approved guidelines. Primary bronchial epithelial cells (pBEC) were obtained from bronchial brushings and cultured under submerged conditions on collagen‐coated flasks (Thermo Fisher) in supplemented bronchial epithelial growth medium (Lonza, Melbourne, Australia). All bronchial brushings were obtained from the same anatomical regions (bronchial generations 4–7) and were used within five passages.

### Isolation of human peripheral blood mononuclear cells

Informed written consent was obtained from all healthy blood donor volunteers and ethical approval was obtained from the local Lothian Research Ethics Committee (AMREC 15‐HV‐013 and CIR 20‐HV‐069). Human monocyte‐derived macrophages (MDMs) were prepared from peripheral blood as described.^60^ Briefly, peripheral blood mononuclear cells (55/70 interface) were isolated from whole blood using isotonic discontinuous PBS/Percoll gradients.

### Culture of human monocyte‐derived macrophages

MDMs were prepared from whole blood by adherence of the monocytes to the tissue culture plates followed by washing off non‐adherent lymphocytes.^61^ Cells were resuspended at 2 × 10^6^ cells mL^−1^ in DMEM supplemented with 10% donor autologous serum (prepared by adding 220 μL of 1 m calcium chloride (CaCl_2_) to 10 mL of platelet‐rich plasma and incubated for 2 h at 37°C). One millilitre per well of the cell suspension was added to a 24‐well tissue culture plate (Corning). Cells were incubated at 37°C/5% CO_2_ for 7 days with a media change at day 3, allowing monocyte differentiation into macrophages.

### *In vitro* stimulation of murine and human macrophages, and human pBECs

BMDMs were seeded at 10^5^ macrophages mL^−1^ in 96‐well plates 24 h prior to incubation with LPS (Serotype O55:B5; 100 ng mL^−1^; Invivogen, San Diego, USA) for 3 h. Cells were incubated with ADS032 compounds or MCC950 at indicated concentrations 1 h prior to stimulation with inflammasome activators (Invivogen), as follows. NLRP3: silica (250 μg mL^−1^; 6 h), nigericin (6 μm; 2 h), monosodium urate crystals (MSU, 250 μg mL^−1^; 6 h) and LPS Serotype 0111:B4 (100 ng mL^−1^, 16 h), transfected with Lipofectamine 2000 (Thermo Fisher). NLRP1: L18‐MDP (100 μg mL^−1^; 16 h) and poly I:C (20 ng mL^−1^; 8 h), transfected with Lipofectamine, talabostat (2.5 μm; 24 h) and anisomycin (2.0 μm; 8 h). AIM: poly (dA:dT) (1 μg mL^−1^), transfected with Lipofectamine. NLRC4: flagellin (20 mg mL^−1^; 6 h), transfected with Lipofectamine. Cultured supernatants were analysed for IL‐1β and TNF‐α (R&D Systems, Minneapolis, USA) by ELISA and lactate dehydrogenase (LDH; Promega, Madison, USA) according to manufacturer's instructions.

### Immunoblot of IL‐1β and caspase‐1

Cultured supernatants derived from BMDMs or PMA‐differentiated THP‐1 macrophages were concentrated by the addition of StrataClean (Agilent Technologies, Mulgrave, Australia). Adhered proteins were released by the addition of Laemelli buffer, 95°C, 5 min. Protein samples were separated by 4–12% SDS‐PAGE (Thermo Fisher), transferred to PVDF resolved by SDS‐PAGE, transferred onto PVDF (EMD Millipore, Cat #IPFL00010), blocked in Odyssey Blocking Buffer (Li‐cor Bioscienes, Lincoln, USA) and incubated with the following primary antibodies overnight: mouse biotinylted IL‐1β and human IL‐1β biotinylted (R&D Systems); mouse anti‐mouse caspase‐1 monoclonal antibody (AdipoGen Life Sciences); and anti‐human Caspase‐1 (D7F10) Rabbit mAb (Cell Signaling Technologies, Danvers, USA).

Membranes were then probed with the appropriate IRDye conjugated secondary antibodies: anti‐rabbit IgG, anti‐mouse IgG (Rockland Immunochemicals, Pottstown, USA). Membranes were scanned using an Odyssey® Infra‐red Imaging System.

### Cross‐linking experiments

Recombinant full‐length human NLRP1 (Creative Biomart, Shirley, USA) or human NLRP3 ΔLRR 1–536 (Cusabio, Houston, USA) (2 μg) were resuspended in lysis buffer (50 mm Tris–HCl pH 7.4, 150 mm NaCl and 0.5% Igepal CA‐630) and incubated with ADS165 or MCC950 where indicated (total volume 40 μL) for 20 min prior to photo‐crosslinking with a UVA fluorescent light box at 365 nm (AnalytikJena, Jena, Germany) for 20 min. ADS165 was then added for a further 20 min and cross‐linked for 20 min. Protein samples were mixed with Laemelli buffer and protein mix separated by SDS‐PAGE, transferred to PVDF membrane and immunoblotted with anti‐PEG (polyeltheylene glycol) (clone RM105; Abcam, Cambridge, UK) or anti‐NLRP1 (clone E3F2U; Cell Signaling Technologies) antibody where indicated.

ASC‐cerulean iBMDMs were seeded at 2 × 10^6^ cells, 20 h prior to incubation with ADS165 (1 mm), ADS167 (1 mm), MCC950 (100 μm) or vehicle control (DMSO) for 30 min prior to exposure to photo‐crosslinking at 365 nm for 30 min. Cells were lysed in lysis buffer supplemented with protease inhibitor (Roche, Basel, Switzerland), 1 mm phenylymethylsulfonyl fluoride (Merck, Bayswater, Australia) and benzonase, centrifuged (14 500 *g*, 4°C, 10 min) to remove debris, and anti‐NLRP3 (clone D4D8T; Cell Signaling Technologies) antibody was added in conjunction with Protein G‐Sepharose (Merck) to precipitate NLRP3 from protein mix. After 2 h rotation (4°C), beads were washed three times with lysis buffer and pellet resuspended in Laemelli buffer. Protein complexes were subjected to SDS‐PAGE, transferred to PVDF membrane (Merck) and immunoblotted with anti‐NLRP3 antibody (clone Cryo‐2; Adipogen Life Sciences, San Diego, USA).

HEK293T cells were seeded at 1 × 10^6^, 20 h prior to ectopic transfection with NLRP1‐Flag (1.25 μg) or empty vector (1.25 μg) and incubated for a further 24 h. Cells were incubated with ADS165 (1 mm), ADS167 (1 mm), MCC950 (100 μm) or vehicle control (DMSO) (Merck) for 30 min prior to exposure to photo‐crosslinking at 365 nm for 30 min. Cells were lysed in lysis buffer supplemented with protease inhibitor (Roche), 1 mm phenylymethylsulfonyl fluoride (Merck) and benzonase, and centrifuged (13 000 rpm, 4°C, 10 min) to remove debris. NLRP1 was precipitated by addition of anti‐Flag (clone M2)‐Sepharose (Merck) beads with rotation for 2 h, and washed three times with lysis buffer. Immunoprecipitated complexes were separated and visualised by SDS‐PAGE and immunoblot with anti‐PEG or anti‐NLRP1 as indicated.

### ASC‐speck bioimaging

ASC‐cerulean‐expressing iBMDMs were incubated with ADS032 or vehicle control (DMSO) for 60 min in serum‐free DMEM prior to challenge with nigericin (6 μm) or silica (250 μg mL^−1^) for 2 or 5 h, respectively, with the addition of Hoechst 33342 (Thermo Fisher) 10 min prior to harvest. Cells were fixed for 30 min in 4% paraformaldehyde (BioRad, Hercules, USA) and nine fields per sample were imaged on a Nikon C1 confocal microscope (Melville, USA) using a 63× oil objective. For ASC‐speck quantification, the imaged fields were analysed as three‐dimensional deconvoluted maximum‐intensity projections of z stacks, with imaging analysis software (Imaris; Bitplane AG, Zurich, Switzerland).

### Animal models

All animal care and experimental procedures complied with the approved guidelines and were approved by the Monash Medical Centre Animal Ethics Committee. Six‐ to eight‐week‐old C57BL/6 male and female mice were maintained in the Specific Pathogen‐Free Physical Containment Level 2 (PC2) Animal Research Facility at the Monash Medical Centre. Wild‐type mice were randomly allocated to treatment groups.

#### LPS‐induced inflammation

Four male and four female mice were treated with ADS032 (200 mg kg^−1^) or vehicle control (0.5% methylcellulose) (Merck) i.p. for 60 min prior to i.p. challenge with LPS (*Escherichia coli* Serotype 0111:B5; 10 mg kg^−1^) for a further 2 h. Mice were euthanised with sodium pentobarbitone i.p. and serum cytokine concentrations were analysed by ELISA for IL‐1β, and TNF‐α, IFN‐γ and IL‐10 by cytokine bead array inflammation kit (Becton Dickinson, Franklin Lakes, USA).

#### Silica‐induced pulmonary inflammation

Male or female mice were lightly anaesthetised with isoflurane prior to intranasal (i.n.) challenge with a total volume of 50 μL of PBS, silica (1 mg kg^−1^) in PBS, silica (1 mg kg^−1^) resuspended in ADS132 (40 mg kg^−1^) in PBS or ADS132 alone for 24 h. Bronchoalveolar lavage fluid (BALF) was obtained following sodium pentobarbitone i.p. administration, by flushing the lungs three times with 1 mL of PBS.

#### Influenza A virus challenge

HKx31 (H3N2) is a high‐yielding reassortment of A/PR/8/34 that carries the surface glycoproteins of A/Aichi/2/1968 (H3N2) responsible for the IAV pandemic of 1968. Virus was grown in 10‐day embryonated chicken eggs by standard procedures and titrated on Madin–Darby canine kidney (MDCK) cells.

Wild‐type male and female C57BL/6 mice were randomised into treatment groups. Mice were lightly anaesthetised with isoflurane and i.n. challenged with 10^5^ plaque‐forming units (PFU) of HKx31 in 50 mL PBS, as previously demonstrated to induce severe disease in C57BL/6 mice.^15^, ^62^ Following infection, mice were treated every 48 h (according to ethically approved guidelines) from either day 1 or day 3 post‐infection as indicated, with ADS032 (20 mg kg^−1^) in PBS (50 μL) i.n. Control mice were treated with PBS (50 μL) alone. Mice were weighed daily and assessed for visual signs of clinical disease, including inactivity, ruffled fur, laboured breathing and huddling behaviour. Animals that lost ≥ 20% of their original body weight or displayed severe clinical signs of disease were euthanised. BALF was immediately obtained following killing of mice as outlined above.

### Quantification of pulmonary inflammation

To detect cytokines, BALF was collected and stored at −80°C. IL‐1β was quantified by ELISA according to the manufacturer's instructions (R&D Systems). Concentrations of IL‐6, moncoyte chemoattractant protein (MCP)‐1, IFN‐γ, IL‐10, IL‐12p70 and TNF‐α proteins were determined by cytokine bead array mouse inflammation kit (Becton Dickinson). For flow cytometric analysis, BALF cells were treated with red blood cell lysis buffer (Merck), and cell numbers and viability were assessed via Trypan blue exclusion using a haemocytometer. BALF cells were incubated with Fc block (2.4G2; eBiosciences), followed by staining with fluorochrome‐conjugated monoclonal antibodies to Ly6C, Ly6G, CD11c and I‐Ab (MHC‐II; BD Biosciences, Franklin Lakes, USA). Neutrophils (Ly6G^+^), airway macrophages (CD11c^+^ I‐Ab^low^), dendritic cells (DC; CD11c^+^ I‐Ab^high^) and inflammatory macrophages (Ly6G^−^ Ly6C^+^) were quantified by flow cytometry, as described previously.^15^ Live cells (propidium iodide negative) were analysed using a BD FACS Canto II flow cytometer (BD Biosciences) and FlowJo software (FlowJo, Irvine, USA). Total cell counts were calculated from viable cell counts performed via trypan blue exclusion.

### Statistics

Statistical analysis was conducted using GraphPad Prism (San Diego, USA). When comparing three or more sets of values, a one‐way ANOVA was used with Tukey's *post hoc* analysis. A Student's *t‐*test was used when comparing two values (two‐tailed, two‐sample equal variance). Survival proportions were compared using the Mantel–Cox log‐rank test.

## Conflict of interest

RED and MAN receive consultancy fees from Adiso Therapeutics Inc. RF, DLF, CKM and AM are employees of Adiso Therapeutics Inc. AJF and AGR are supported by an Adiso Therapeutics Inc research grant to the University of Edinburgh Centre for Inflammation Research. RED, MAN, RF and CKM have patent ownership, while CKM and AM retain stock ownership in Adiso Therapeutics.

## Author contributions

**Callum AH Docherty:** Data curation; formal analysis; investigation; validation; writing – review and editing. **Anuruddika J Fernando:** Data curation; formal analysis; methodology; writing – review and editing. **Sarah Rosli:** Investigation; resources. **Maggie Lam:** Investigation; resources; writing – review and editing. **Roland E Dolle:** Conceptualization; methodology; resources. **Manuel A Navia:** Conceptualization; methodology; resources. **Ronald Farquhar:** Conceptualization; funding acquisition; investigation; resources; supervision. **Danny La France:** Conceptualization; resources. **Michelle D Tate:** Investigation; methodology; resources; supervision; writing – review and editing. **Christopher K Murphy:** Conceptualization; formal analysis; funding acquisition; project administration; writing – original draft; writing – review and editing. **Adriano G Rossi:** Formal analysis; investigation; project administration; supervision; writing – review and editing. **Ashley Mansell:** Conceptualization; data curation; formal analysis; funding acquisition; investigation; methodology; project administration; supervision; validation; writing – original draft; writing – review and editing.

## Supporting information


Supplementary figures 1–5

## Data Availability

The data that support the findings of this study are available from the corresponding author upon reasonable request.
