# Supplementary material for: A novel dual NLRP1 and NLRP3 inflammasome inhibitor for the treatment of inflammatory diseases
Source: Clin Transl Immunology. 2023 Jun 22;12(6):e1455. doi: 10.1002/cti2.1455 (PMC10288073; doi:10.1002/cti2.1455)
Supplement: Supplementary file 1 — Supplementary figures 1–5 [file CTI2-12-e1455-s001.pdf]

SUPPORTING INFORMATION

A novel dual NLRP1 and NLRP3 inflammasome inhibitor for the treatment of inflammatory diseases

Running Title: ADS032 - a potential NLRP1 and NLRP3 therapeutic

Callum AH Docherty[1,2,a], Anuruddika J Fernando[3,a], Sarah Rosli[1,2], Maggie Lam[1,2], Roland E Dolle[4], Manuel A Navia[5], Ronald Farquhar[6], Danny La France[6], Michelle D Tate[1,2], Christopher K Murphy[6], Adriano G Rossi[3], **Ashley Mansell**[1,2,6]

[1]Centre for Innate Immunity and Infectious Diseases, Hudson Institute of Medical Research, Clayton, VIC, Australia

[2]Department of Molecular and Translational Sciences, Monash University, Clayton, VIC, Australia

[3] University of Edinburgh Centre for Inflammation Research, Queen's Medical Research Institute, Edinburgh BioQuarter, Edinburgh, UK

[4]Department of Biochemistry and Molecular Biophysics, Washington University School of Medicine, 660 S. Euclid Ave. St. Louis, Missouri 63110, USA

[5] Hub-Bio Strategic Advising, Lexington, MA 02420, USA

[6]Adiso Therapeutics, 530 Virginia Road, Suite 300, Concord, MA 01742, USA

[a]Equal Contribution

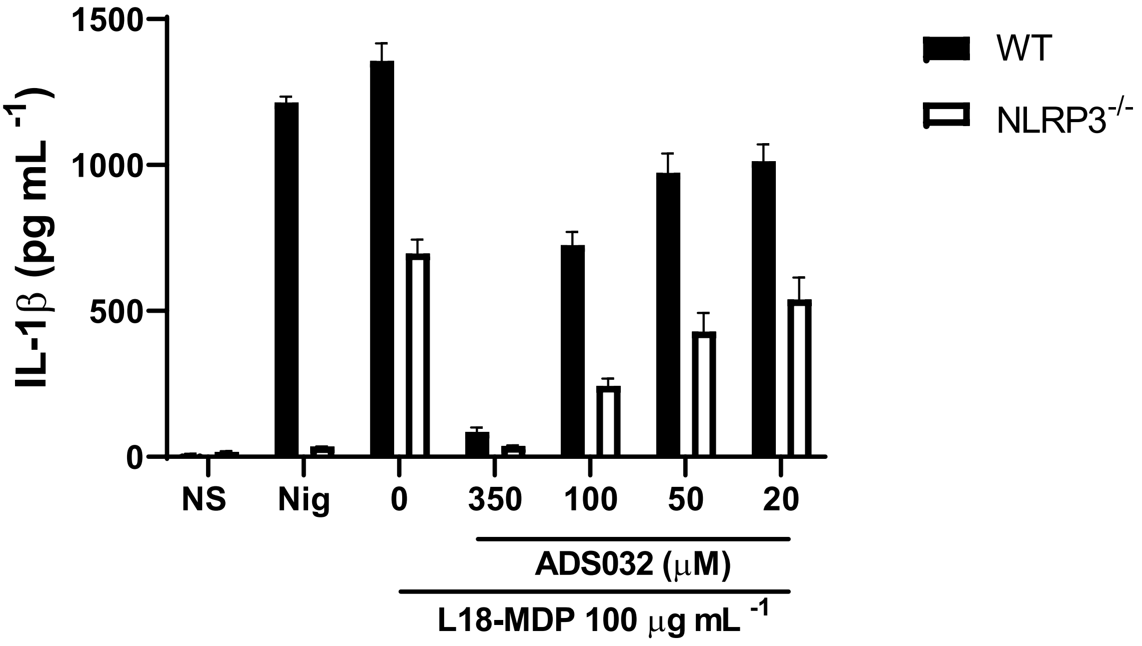

Supplementary Figure 1. L18-MDP-induced IL-1β secretion is not dependent upon NLRP3.

Immortalized bone marrow-derived macrophages (iBMDMs) were seeded at $4x10^5$ mL $^{-1}$ prior to priming with LPS (100 ng mL $^{-1}$) for 3 h. Media was removed 60 min prior to challenge and replaced with serum-free media containing ADS032 (3.5-350 μM) as indicated, or DMSO control (0.8% v/v). Macrophages were stimulated with either Nigericin (6 μM) or silica (250 μg mL $^{-1}$) as indicated for 120 or 360 min, respectively. Cellular supernatants were analyzed for secreted IL-1β by ELISA. Data shown is representative of three independent experiments carried out in triplicate and presented as the mean ± SEM.

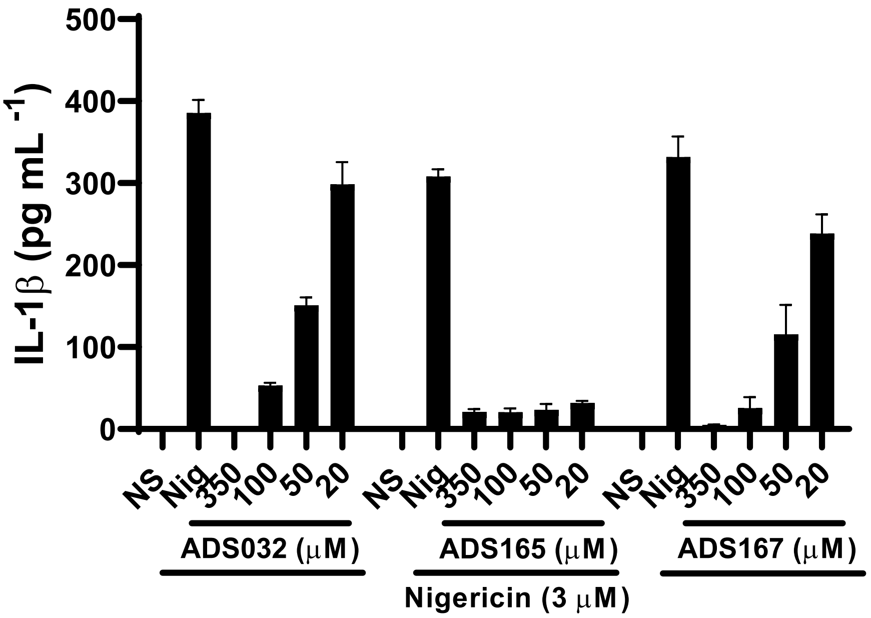

Supplementary Figure 2. Photoactivatable ADS032 analogs also inhibit NLPR3 inflammasome activity.

(iBMDMs) were seeded at $4x10^5$ mL $^{-1}$ prior to priming with LPS (100 ng mL $^{-1}$) for 3 h. Media was removed 60 min prior to challenge and replaced with serum-free media containing ADS032 (3.5-350 μM) as indicated, or DMSO control (0.8% v/v). Macrophages were stimulated with Nigericin (3 μM) for 120 min. Cellular supernatants were analyzed for secreted IL-1β by ELISA. Result shown is representative of three independent experiments and presented as the mean ± SEM.

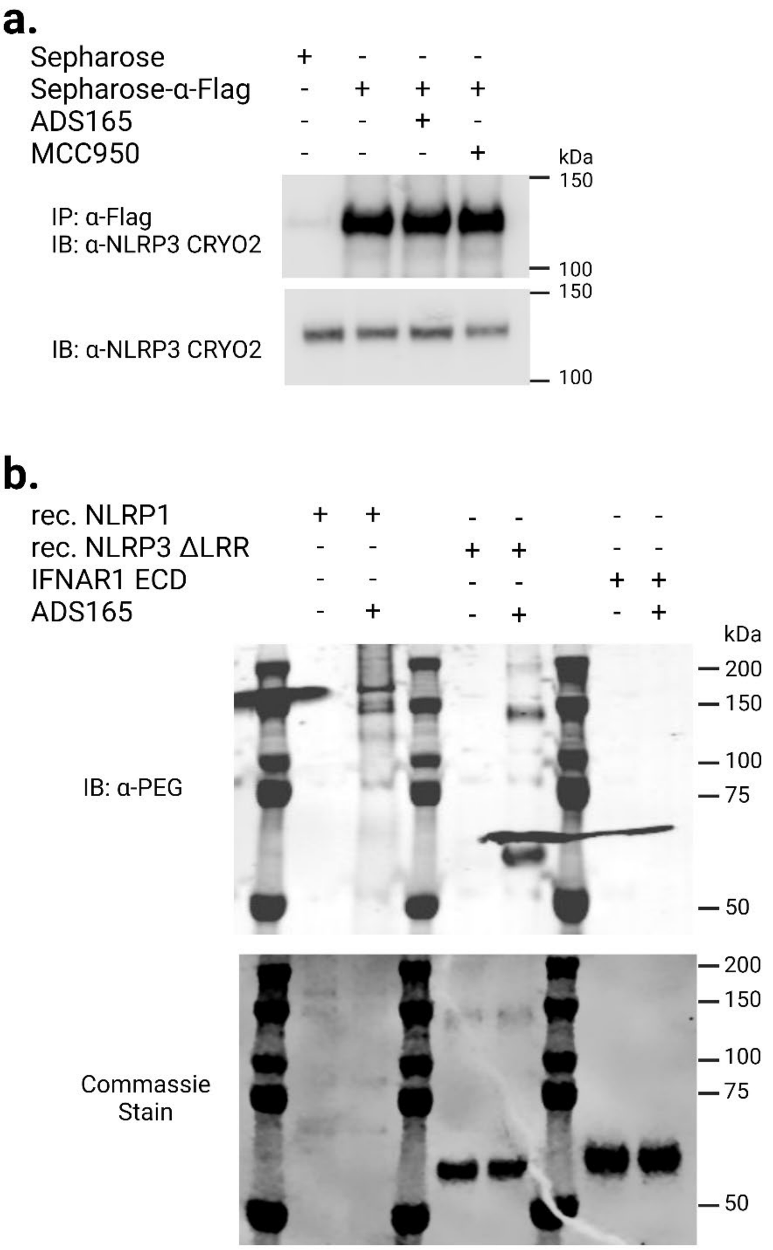

Supplementary Figure 3. Photoactivatable ADS165 specifically binds NLRP1 and NLRP3. (**a**) iBMDM cells stably expressing NLRP3-Flag were treated for 30 min with ADS165 (1 mM) or MCC950 (50 μM) for 30 min and irradiated with UV 365 nm for 30 min. Cells were lysed with buffer and immunoprecipitation (IP) of NLRP3 performed with α-Flag (M2)-Sepharose beads. Levels of precipitated (IP) and total cellular lysate of NLRP3 were determined by immunoblotting with α-NLRP3 (Cryo-2) antibody. (**b**) Recombinant NLRP1 NLRP3 and INFAR1 extracellular domain (ECD) (2 μg) were coincubated where indicated with ADS165 for 20 min and then irradiated with UV 365 nm for a further 20 min. Proteins was separated by SDS-PAGE and stained with Commassie Blue stain for total protein (lower panel) or transferred to PVDF membrane and immunoblotted (IB) with anti-PEG to visualize ADS165-linked protein (upper panel). Results presented are representative of three independent experiments.

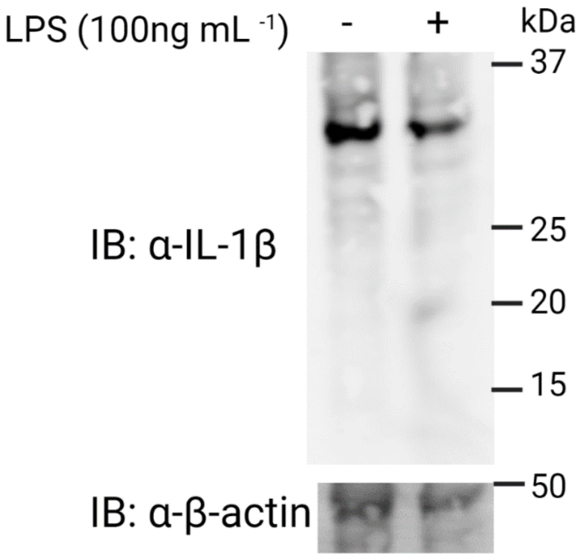

Supplementary Figure 4. Primary human bronchial epithelial cells constitutively express pro-IL-1β.

Bronchial epithelial cells obtained from normal patients were treated or not with LPS (100 ng mL^{-1}) for 3 h prior. Cell lysates were examined for IL-1β expression by immunoblot with α-IL-1β antibody. β-Actin staining was conducted to demonstrate protein loading. Data shown is representative of 2 experimental replicates.

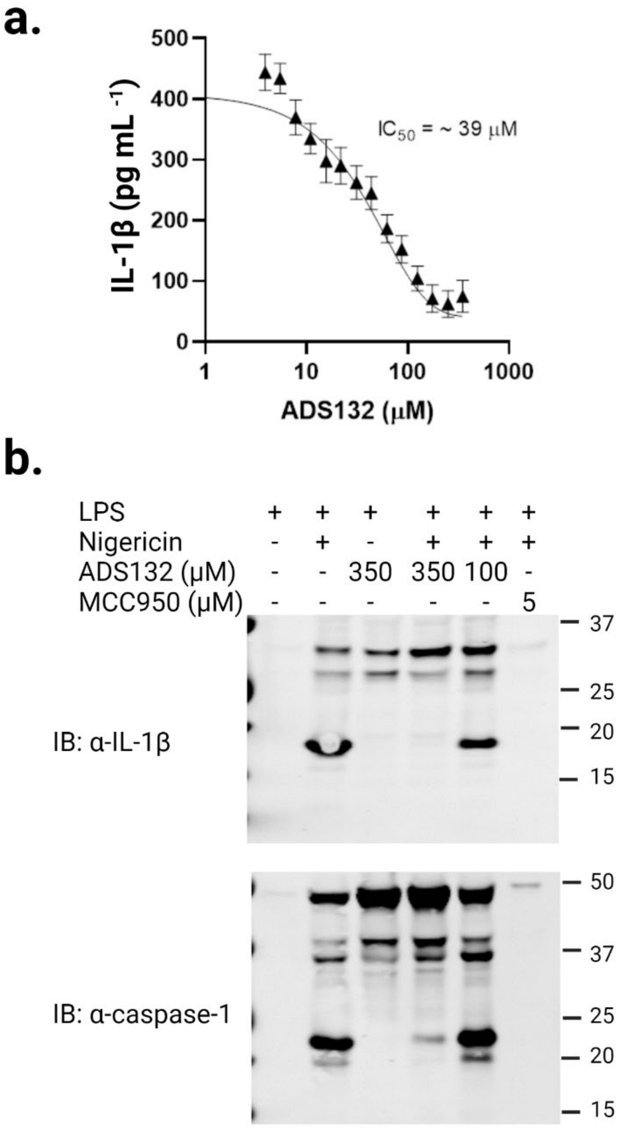

Supplementary Figure 5. ADS132 inhibits NLRP3 inflammasome activity.

(**a**) Immortalized bone marrow-derived macrophages (iBMDMs) were seeded at 4×10^5 mL $^{-1}$ prior to priming with LPS (100 ng mL $^{-1}$) for 3 h. Macrophages were treated with a range of ADS032 concentrations for 60 min prior to challenge with Nigericin for 120 min. Cellular supernatants were analyzed for secreted IL-1β by ELISA. Data shown are pooled data of three independent experiments carried out in triplicate and presented as the mean ± SEM. Non-linear regression analysis was performed, and the curve of the log [M] ADS032 versus the normalized response (variable slope) is presented. (**b**) BMDMs derived from WT mice were primed with LPS (100 ng mL $^{-1}$) for three hours prior to treatment with ADS132 (350-100 μM) or MCC950 (5 μM) where indicated for 60 mins. Macrophages were then challenged with nigericin (6 mM) for 2 h. Supernatants were separated by 4-12% SDS-PAGE and immunoblotted (IB) with indicated antibodies. Results shown are representative of two independent experiments.
